# Chilling, irradiation and transport of male *Glossina palpalis gambiensis* pupae: Effect on the emergence, flight ability and survival

**DOI:** 10.1371/journal.pone.0216802

**Published:** 2019-05-14

**Authors:** Souleymane Diallo, Momar Talla Seck, Jean Baptiste Rayaissé, Assane Gueye Fall, Mireille Djimangali Bassene, Baba Sall, Antoine Sanon, Marc J. B. Vreysen, Peter Takac, Andrew Gordon Parker, Geoffrey Gimonneau, Jérémy Bouyer

**Affiliations:** 1 Centre International de Recherche-Développement sur l’Élevage en Zone Subhumide–CIRDES,Bobo–Dioulasso, Burkina Faso; 2 Laboratoire d’Entomologie Fondamentale et Appliquée, Unité de Formation et de Recherche en Sciences de la vie et de la Terre, Université Ouaga I Pr Joseph Ki Zerbo, Burkina Faso; 3 Institut Sénégalais de Recherches Agricoles, Laboratoire National d’Elevage et de Recherches Vétérinaires, Service de Bio-écologie et Pathologies Parasitaires, BP, Dakar—Hann, Sénégal; 4 Direction des Services Vétérinaires, Dakar, Sénégal; 5 Insect Pest Control Laboratory, Joint FAO/IAEA Programme of Nuclear Techniques in Food and Agriculture, Vienna, Austria; 6 Institute of Zoology, Slovak Academy of Sciences, Bratislava, Slovakia and Scientica, ltd, Hybesova 33, Bratislava, Slovakia; 7 CIRAD—Département Systèmes Biologiques - UMR 17 Intertryp CIRAD/IRD, Campus International de Baillarguet, Montpellier, France; 8 CIRAD—Département Systèmes Biologiques - UMR ASTRE CIRAD/INRA, Montpellier, France; Liverpool School of Tropical Medicine, UNITED KINGDOM

## Abstract

**Background:**

The sterile insect technique (SIT) requires mass-rearing of the target species, irradiation to induce sexual sterility and transportation from the mass-rearing facility to the target site. Those treatments require several steps that may affect the biological quality of sterile males. This study has been carried out to evaluate the relative impact of chilling, irradiation and transport on emergence rate, flight ability and survival of sterile male *Glossina palpalis gambiensis*.

**Results:**

Chilling, irradiation and transport all affected the quality control parameters studied. The emergence rate was significantly reduced by long chilling periods and transport, i.e. from 92% at the source insectary in Burkina Faso to 78% upon arrival in Senegal. Flight ability was affected by all three parameters with 31% operational flies lost between the production facility and the destination site. Only survival under stress was not affected by any of the treatments.

**Conclusion:**

The chilling period and transport were the main factors that impacted significantly the quality of sterile male pupae. Therefore, in the operational programme, the delivery of sterile male pupae was divided over two shipments per week to reduce the chilling time and improve the quality of the sterile males. Quality of the male pupae may further be improved by reducing the transport time and vibrations during transport.

## Introduction

Tsetse flies (Diptera: Glossinidae) are confined to sub-Saharan Africa, and are the cyclical vectors of trypanosomes, the causative agents of African animal trypanosomosis (AAT) or nagana in animals and human African trypanosomosis (HAT) or sleeping sickness in humans[1]. In the agricultural sector, the presence of tsetse flies limits the exploitation of fertile lands in the more than 10 million km^2^ and about 50 million cattle and tens of millions of small ruminants are permanently at risk of becoming infected with AAT [2]. This results in estimated annual economic losses of USD 600 million to 1.2 billion for the livestock sector [3] and overall annual losses of USD 4.75 billion for the global livestock and crop production areas [4]. Therefore, tsetse and trypanosomosis constitute a major constraint to livestock production and are one of the main factors preventing the development of more sustainable and productive livestock systems in much of sub-Saharan Africa. The absence of vaccines and high cost of disease treatment associated with the development of resistance by the parasites to the used trypanocidal drugs [5] make vector management a more sustainable option for the management of the disease [6].

Currently, several control tactics are available to manage populations of tsetse flies, i.e. ground fogging of insecticides, the sequential aerosol technique (SAT–aerial spraying of ultra-low-volume insecticides), bait techniques such as insecticide-impregnated targets or insecticide-treated animals, the use of tsetse traps and the sterile insect technique (SIT) [7]. In contrast to the majority of control tactics that aim to kill tsetse flies, the SIT aims at reducing the reproduction rate of a wild tsetse fly population by area-wide inundative releases of sterile male insects of the same species. The sterile male flies will compete with the wild males population for mating with wild females. A mating of a sterile male with a virgin wild female results in no offspring. As a result of the reduction in the replacement rate of the population, the density of the wild population in the next generation will be decreased.

The SIT has been used with success to suppress or eradicate populations of several insect pests of economic and sanitary importance [8]. For tsetse flies, the SIT was used to eliminate populations of *Glossina palpalis gambiensis* Vanderplank, *Glossina morsitans submorsitans* Newstead and *Glossina tachinoides* Westwood from 3,000 km^2^ in Burkina Faso [9], and *Glossina palpalis palpalis* (Robineau-Desvoidy) from 1500 km^2^ in Nigeria [10]. Although these projects succeeded in eradicating the target populations, they were not conducted in the context of an area-wide integrated pest management (AW-IPM) [8,11] approach and the cleared areas were re-invaded after the programs ended. The SIT was, however, successfully applied following AW-IPM principles to create a sustainable zone free of *Glossina austeni* Newstead (Diptera: Glossinidae) on Unguja Island of Zanzibar, United Republic of Tanzania (1994–1997)[12]. This project confirmed the feasibility of integrating releases of sterile males with other suppression methods to create sustainable tsetse-free areas.

The Government of Senegal initiated in 2005 a program called “Projet d’éradication des mouches tsé-tsé dans les Niayes” [13] with the objective to sustainably remove a population of *G*. *p*. *gambiensis* from a 1,000 km^2^ area in the Niayes region neighbouring the capital, Dakar. A feasibility study confirmed the isolated nature of the tsetse population in the Niayes, and hence, indicated the potential to create a sustainable zone free of *G*. *p*. *gambiensis* in the Niayes [13,14]. Therefore, the Government of Senegal opted for an AW-IPM approach that included an SIT component. Since 2013, the Centre International de Recherche-Développement sur l’Elevage en zone Subhumide (CIRDES) in Bobo-Dioulasso, Burkina Faso, the Slovak Academy of Sciences (SAS) in Bratislava, Slovakia, and the FAO/IAEA Insect Pest Control Laboratory (IPCL) in Seibersdorf, Austria have been shipping sterile male pupae to Senegal. The adult sterile male flies emerged in an insectary located at the Institut Sénégalais de Recherches Agricoles (ISRA), Dakar and were used for release in the target area. Since 2017, the Insectary of Bobo Dioulasso (IBD) in Bobo Dioulasso, Burkina Faso has also provided sterile male pupae to the programme.

The supply of sterile flies requires several important treatments in the production facilities that may have reduced the quality of the sterile male pupae at the delivery site in Senegal, such as chilling, handling, irradiation, and long-distance transport. Preliminary observations already indicated that the flight ability of sterile male *G*. *p*. *gambiensis* that emerged from pupae sent to the ISRA insectary was lower as compared with flies that emerged at the CIRDES and SAS facilities [15]. This reduction in quality could be related to the chilling and irradiation treatments and the long-distance transport of the sterile male pupae. This is of prime importance as the quality of the released sterile males remains one of the most crucial prerequisites for the success of AW-IPM programs that have an SIT component.

In this study, an analysis was made of the effect of various treatments of the pupae such as chilling, irradiation and transport seperately and in combination on the quality of the emerging adult flies. Three biological parameters were used to quantify the impact, i.e. i) adult emergence, ii) flight ability, and iii) survival of the flyers under starvation. The study followed the procedures as described in the quality control protocol by Seck et al.[15].

## Materials and methods

### Insectaries

Samples of male pupae that were destined for the programme in Senegal were used in this study that was conducted between between January 2015 and June 2016. The pupae were produced in two mass-rearing facilities, i.e. the CIRDES and the SAS. Quality control procedures were carried out in these facilities for each shipment as well as in the receiving institute (ISRA) in Senegal. The colonies in all three insectaries were maintained under the same environmental conditions: 24–25°C, 75 ± 5% rH and 12:12 light/dark photoperiod.

### Biological material

The *G*. *p*. *gambiensis* pupae used in this study originated from the BKF strain that is being maintained at the CIRDES and the SAS. The colony was established in 1972 at Maison Alfort (France) with pupae collected from Guinguette (Bobo-Dioulasso) and in 1975, the colony was transferred to the Centre de Recherche sur la Trypanosomiase Animale (CRTA) (later renamed CIRDES). The colonies were maintained using standard rearing procedures and were offered blood meals using an *in vitro* silicon membrane feeding system. The blood was collected from slaughtered cattle from the local abattoir and then irradiated in a ^137^Cs source with a dose of 500 Gy [16].

In 2009, 8000 pupae of this colony were shipped to the Insect Pest Control Laboratory of the Joint FAO/IAEA Programme of Nuclear Techniques in Food and Agriculture, Seibersdorf, Austria to establish a colony for research purposes to support the eradication programme in the Niayes [17,18]. The IPCL provided seed material from this colony to the SAS to establish a colony to supply additional pupae for the Senegal project.

### Chilling and irradiation

At the CIRDES and SAS insectaries, pupae destined for Senegal, were collected daily and incubated at 25±1°C and 75±5% RH. After most of the female flies had emerged, the remaining male pupae were chilled at 4°C to prevent emergence. Pupae were irradiated under chilled conditions (4–6°C) [19] in a ^137^Cs source for 24 minutes and 30 seconds at the CIRDES obtaining a dose of 110 Gy. The SAS pupae were shipped under chilled conditions to the IPCL where they were irradiated in a ^60^Co irradiator (Gammacell 220, Nordion Ltd, Ottawa, Canada; dose rate of 131.4 Gy.min^-1^), or in a 150 kV X-ray irradiator (Rad Source RS2400; dose rate of 14.3 Gy.min^-1^).

### Effect of chilling, irradiation and transport of male pupae on performance parameters of emerged adults

The objective of the quality control is to evaluate the quality of sterile males, measured by the percentage of operational flies corresponding to the percentage of flies escaping the flight device [15]. Here, these quality control parameters were used to measure the impact of various treatments (chilling, irradiation, transport) alone and in combination on the emergence rate and the flight ability of the emerged males.

Batches of 50 pupae were collected from the pupae that were destined for the programme in Senegal and were subjected to the following treatments at the CIRDES:

CIRDES_A0: the control pupae not subjected to chilling or irradiation. These pupae were continuously maintained under normal colony conditions. The control group consisted of 116 replicates of each 50 pupae.
CIRDES_A1: pupae subjected to chilling (8 ± 2°C) for 24 to 72 hours (no irradiation), extracted from the various batches prepared for the programme in Senegal. The A1 group consisted of 110 replicates of each 50 pupae.
SAS_A1: pupae subjected to chilling (8 ± 2°C) for 24 to 72 hours (no irradiation). The A1 group consisted of 146 replicates of each 50 pupae.
CIRDES_A2: pupae subjected to chilling (8 ± 2°C) for 24 to 72 hours followed by an irradiation treatment. The A2 group consisted of 113 replicates of each 50 pupae.
CIRDES_A3: pupae subjected to chilling (8 ± 2°C) for 24 to 72 hours followed by an irradiation treatment and a second chilling (8 ± 2°C) for 48 hours. The A3 group consisted of 114 replicates of each 50 pupae.
CIRDES_A4: pupae that were part of each contingent of shipments to Senegal. These pupae accumulated all the treatments of A3 in addition to the transport (by road and air) in an insulated box to maintain the temperature at 10 ± 2°C. The A4 group consisted of 468 replicates of each 50 pupae. The shipping and handling procedures of these pupae were as follows:

Irradiated pupae were placed in Petri dishes and packed in insulated boxes containing phase change material packs (S8, PCM Products Limited, Cambridgeshire, U. K.) to maintain the temperature at 10 ± 2°C during the entire shipping period. The box size and the number of S8 packs used were adjusted to the number of pupae shipped [19]. A temperature and humidity Hobo data logger (model EL-USB-2) was added to the shipping box during the packing at source insectaries to record the temperature and relative humidity inside the insulated box every 5 minutes. Pupae from the CIRDES were transported from Bobo-Dioulasso to Ouagadougou using public busses and shipped between Ouagadougou and Dakar with a courier service (DHL) that was using commercial aircraft. The average transport and chilling time for pupae from the CIRDES was between 72 and 84 hours. The pupae were maintained at 8 ± 2°C for 24–48 hours at the CIRDES and at 10 ± 2°C for ±36 hours during the transport to Dakar. The pupae from SAS were incubated and shipped to ISRA at 10°C [15,19].

### Quality control protocols

For each treatment, a standard quality control protocol was applied. Briefly, the 50 pupae were put in Petri dishes under ~1cm of sand mixed with a fluorescent dye (DayGlo) (0.5g dye/200g of sand), to mimic the natural emergence conditions in the soil and to allow discrimination of the sterile male flies from wild flies during entomological monitoring in AW-IPM programmes that have an SIT component. The Petri dish was put in a flight cylinder, i.e. a PVC tube 10 cm high and 8.4 cm in diameter [15]. The inner wall of the cylinder was coated with unscented talcum powder to prevent the flies from crawling out. Flies flying out of the tube were considered as “operational flies” (i.e. available for the SIT).

The survival of the sterile males that escaped the flight cylinder was assessed under stress conditions (no blood meal). Every morning, the emerged flies were collected and transferred to standard fly holding cages. The flies emerged on a given day were pooled in one cage. Dead flies were counted daily and removed from the cages.

### Data analysis

Data analysis was performed using the R software version 3.5.1 [20]. Data were analysed using binomial linear mixed effects models using the package lme4 [21], with the emergence rate or rate of flyers as the response variables, the batch origin (CIRDES or SAS) and treatment type (A0 to A4) as fixed effects and the date of arrival at ISRA as a random effect[21–23]. A Gaussian linear mixed effect model was used to analyse the mean survival under starvation, with the same fixed and random effects as in the previous models.

## Results

### Emergence rate

**Fig 1:**
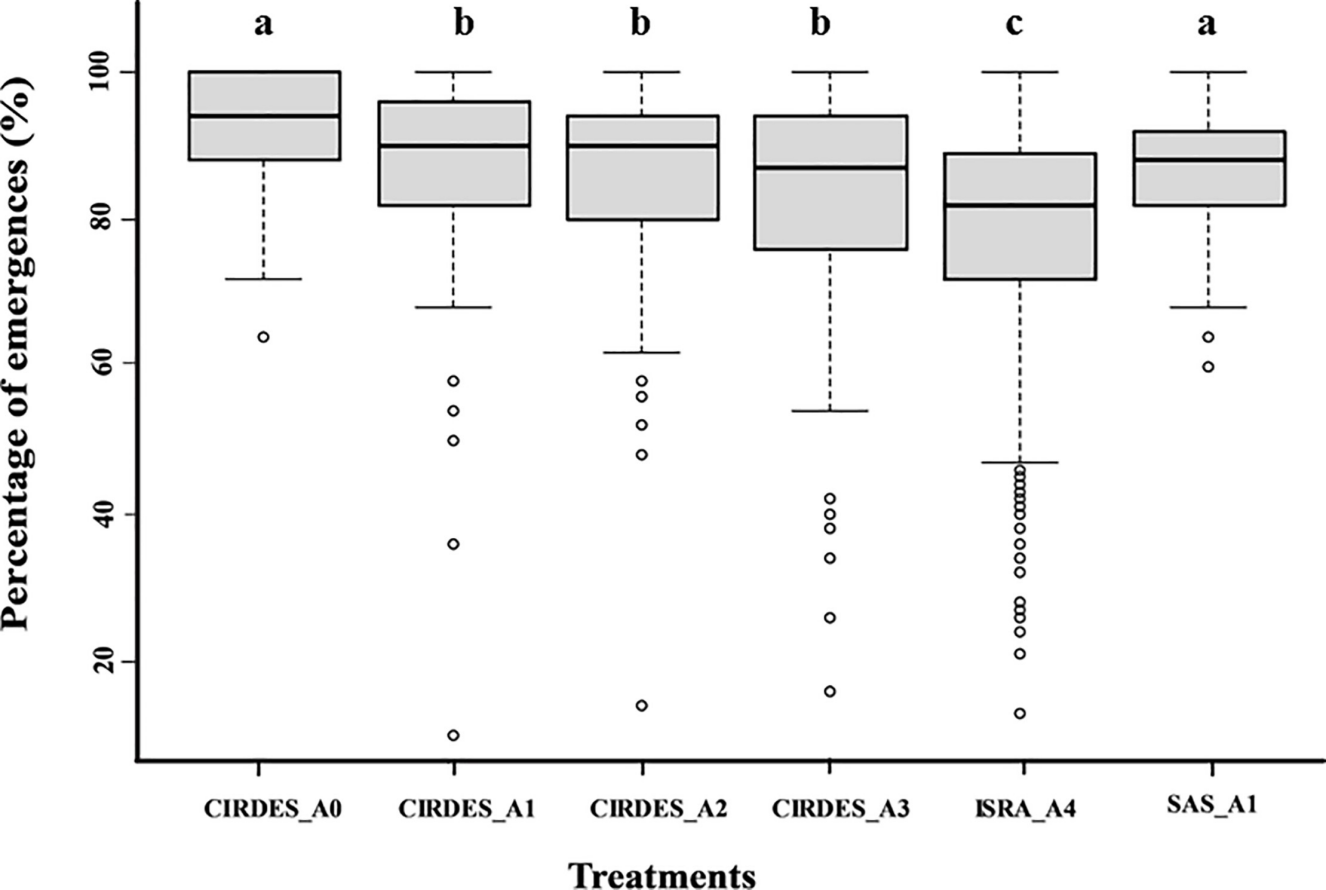
Percentage of emergence according to the treatment and sites. Boxes extend between the 25^th^ and 75^th^ percentile. A thick line denotes the median. The whiskers extend up to the most extreme values and white circles represent outlier data. Different letters indicate significant differences (*P* < 0.05).

The mean emergence rate observed for the five treatments ranged between 92 ± 8% for A0 and 78 ± 15% for A4 with an overall mean at 83 ± 14% (Fig 1). The model results showed that at the CIRDES, the first and second chilling (A1 and A3) significantly reduced adult emergence (*P* < 0.001) whereas there was no further reduction after irradiation (A2) (*P* > 0.05; S1 Table). The emergence rate of A1 treatment was significantly higher for the CIRDES pupae as compared with the SAS pupae (*P* < 0.001), although the mean values were very similar (Fig 1). The transport also significantly reduced the rate of emergence (A4 pupae) (*P* < 0.001) as compared with the A3 pupae.

### Operational flies

**Fig 2:**
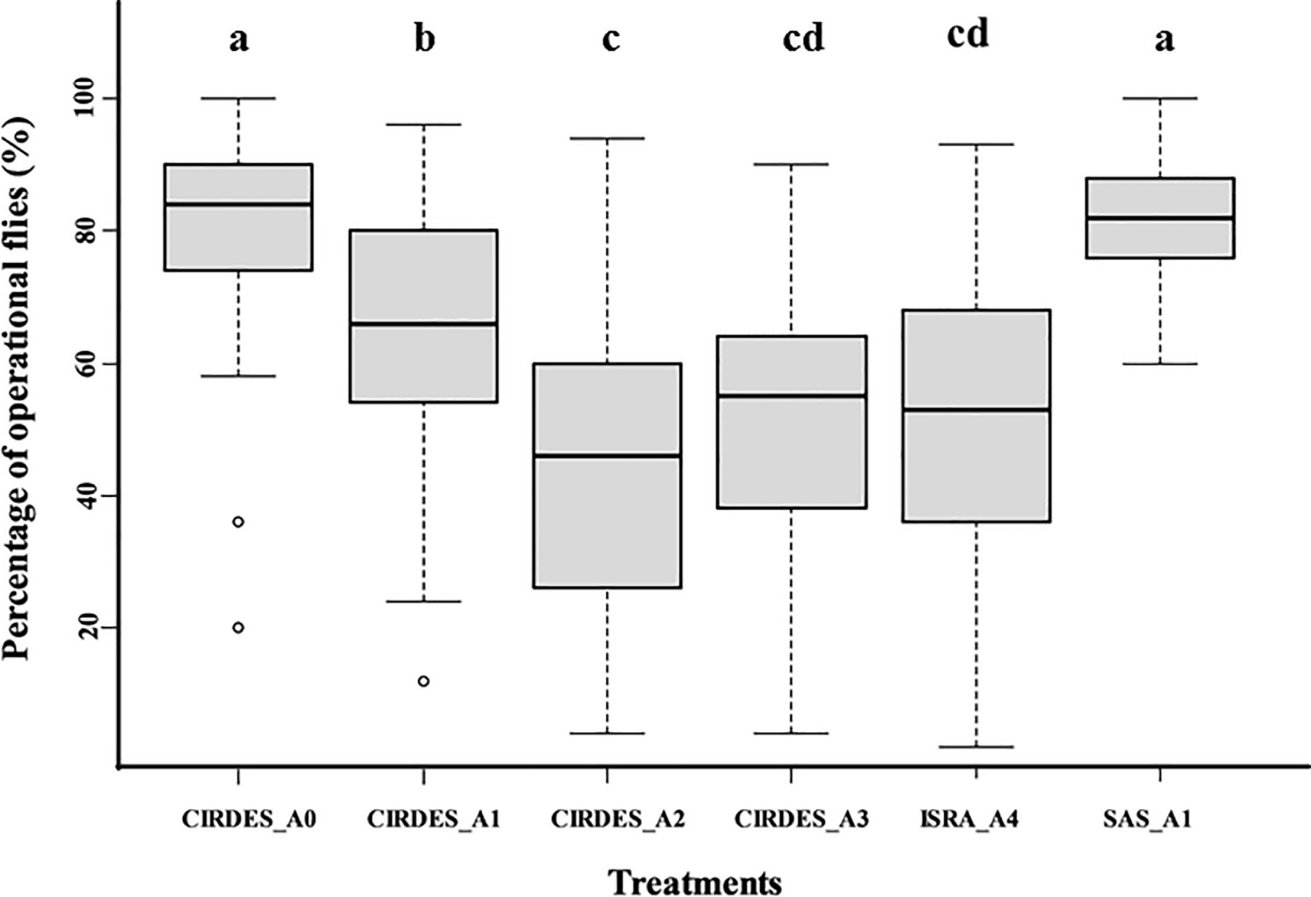
Percentages of operational flies (%) according to treatment (A0 to A4) and site where the test was performed. Boxes extend between the 25^th^ and 75^th^ percentile. A thick line denotes the median. The whiskers extend up to the most extreme values, and white circles represent outlier data. Different letters highlight significant differences (P < 0.05).

The control pupae A0 and the A1 SAS pupae had the highest mean percentage of operational flies (82 ± 13%), whereas A4 pupae had the lowest percentage of operational flies (51 ±21%) (Fig 2). Results of the binomial mixed effects model showed that the first chilling (A1) and the irradiation (A2) both significantly reduced the rate of operational flies at the CIRDES as compared with the control (A0) (*P* < 0.001; S2 Table). The rate of operational flies originating from the A1 pupae was significantly better for the SAS flies than the CIRDES flies (*P* < 0.001). Interestingly, the second chilling (A3) significantly increased the rate of operational flies at the CIRDES as compared with males emerged from the A2 treatment (*P* < 0.001). Finally, the transport from Burkina Faso to Senegal also significantly reduced the rate of operational flies (*P* < 0.001) as compared with males emerged from the A3 treatment (Fig 2).

### Survival under stress

**Fig 3:**
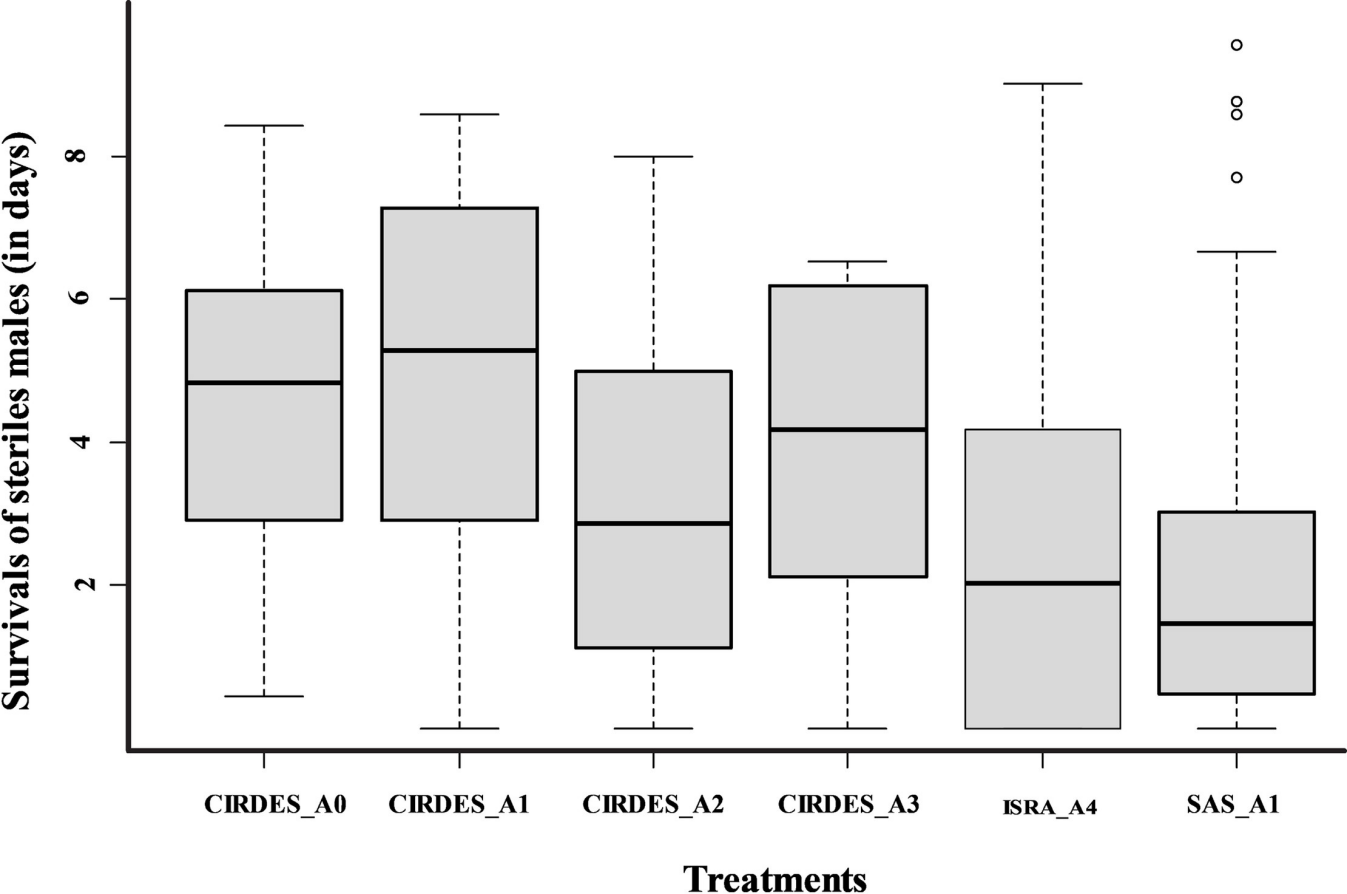
Boxplots of the survival of sterile males (in days) monitored under starvation conditions during the quality test for the four treatments (A0 to A4) and the three insectaries where the tests were carried out. Boxes extend between the 25^th^ and 75^th^ percentile. A thick line denotes the median. The whiskers extend up to the most extreme values, and white circles represent outlier data. Different letters indicate significant differences (P < 0.05).

Fly survival rates under starvation were very similar between treatments ranging from 4 to 5 days, except for A1 SAS that showed a lower mean survival rate of 2.3 days (*P* < 0.005, Fig 3). Model results showed that at the CIRDES none of the treatments had an impact on survival (*P* > 0.05; S3 Table). Finally, the survival of males from the A4 batch at the ISRA was significantly better than the A3 batch at the CIRDES (*P* = 0.006).

## Discussion

The sterile insect technique depends on the mass-production of sterile male insects of good biological quality to be released into the target wild populations. The tsetse eradication programme in the Niayes of Senegal is implemented following an AW-IPM approach with an SIT component and aims at eradicating the native *G*. *p*. *gambiensis* populations in that region [24]. The sterile insects are provided to the project as irradiated pupae that are being transported from four production centres (two in Burkina Faso, one in Slovakia and Austria) under chilled conditions. Handling, irradiation, chilling and transport may affect the quality of the adult male flies and their performance upon arrival in Dakar. Previous studies have already shown that the emergence rate and the quality of the sterile males are lower in the Dakar insectary than in the production centres [15,19]. Here we used the quality control protocols developed by Seck et al. to assess the impact of chilling, irradiation and transport of the male pupae on i) adult emergence, ii) flight ability of the emerged males, and iii) survival of the male flyers under starvation.

### Emergence rate

The emergence rate of pupae was negatively affected by all handling processes, decreasing from 92±8% for A0 pupae at the CIRDES to 78±15% for A4 pupae at the ISRA. Both chilling events (A1 and A3) significantly reduced the number of emerging flies whereas irradiation (A2) did not. This result is in contrast with previous studies on the chilling effect on tsetse fly pupae. Mutika et al. [17] worked with the same *G*. *p*. *gambiensis* BKF strain and observed that emergence of 28-day old male pupae after storage at low temperature (10°C) for 3, 5, or 7 days was similar to those kept under standard colony conditions. Similar results were obtained for *G*. *morsitans* pupae maintained at 12°C for two weeks [25]. Our results of the current study indicate that the low temperature used during the experimental period affected tsetse emergence rate significantly and the chilling period must therefore be kept as short as possible. It must be noted that our results were obtained with a temperature that was on average 2 to 4°C lower than the previous studies, which might partially explain this discrepancy. On the other hand, irradiation had no significant negative effect on pupal emergence. Similar results were observed for *G*. *tachinoides* pupae irradiated with 120 Gy on day 28 post larviposition [26]. and for *G*. *p*. *gambiensis* pupae irradiated with 110 Gy on days 25, 27 and 29 post larviposition [26]. These results were expected since several studies in the past have aimed at optimising the irradiation process in order to induce near 100% sterility while minimising somatic damage [26–28].

The transport to Dakar also significantly reduced fly emergence. It must be noted however, that during shipment, pupae are exposed to uncontrolled factors such as vibrations or mechanical shocks that are absorbed by pupae and may affect emergence rate. This is frequently observed in butterfly chrysalides shipped to tropical butterfly greenhouses (Bouyer, unpublished data). Therefore, solutions should be tested such as the use of cotton wool, sawdust or vermiculite to cushion the mechanical shocks or vibrations during transport. Moreover, adult emergence may also be affected by excessive temperatures or inappropriate relative humidity during the rearing process [29,30].

Although adult emergence was negatively affected by the various treatments, the emergence rate improved with time during project implementation. Indeed, Pagabeleguem et al.[19] observed an emergence rate of pupae received at the ISRA of 74 ± 13.9% for shipments between January 2011 and 2014 against 78 ± 15% in this study. Moreover, our results were not different from those of Mutika et al.[17], who observed emergence rates between 76 to 91% for irradiated pupae chilled for 5 days.

### Operational flies

The rate of operational flies was also affected by all pupal treatments, decreasing from 82 ± 13% for A0 pupae to 51 ± 21% for A4 pupae. Eighteen percent and 19% of operational flies were lost due to the first two treatments A1 and A2, respectively, leading to a cumulative loss of 47% after chilling and irradiation.

These results contrast with several studies that showed that storage of *Glossina p*. *gambiensis Glossina pallidipes* and *Haematobia irritans* mature pupae at low temperature (up to 5 days between 7–10°C) had no detrimental effects on male emergence, flight ability or survival[17,30,31]. The chilling period for A1 and A2 pupae were comparatively short (only 24 to 72 hours at 8°C) and such losses could not be explained by the cold exposure only. Reducing the chilling period could be one option to improve these results.

Moreover, the percentage of operational flies was significantly better for SAS A1 pupae compared to CIRDES A1 pupae. This difference could be explained by the different chilling durations between the SAS and the CIRDES pupae, given that CIRDES pupae were chilled for two to three days before their irradiation whereas it is only one to two days for the SAS pupae (all tested pupae were sampled from pupae batches prepared for the Senegal programme). Therefore, chilling duration and temperature seems to be the two most important parameters that must be surveyed continuously in order to improve fly quality. Although irradiation (A2) also affected the percentage of operational flies, it may be due to the irradiation handling process instead of the radiation dose itself. Earlier studies have examined the effect of radiation dose on quality of pupae and they found that there is no effect on biological quality of pupae when the irradiation is done close to emergence with dose between 110 and 120 Gy [26,27].

Interestingly, the second chilling event (A3) led to a significant “recovery” of the percentage of operational flies at the CIRDES compared to the A2 treatment and this effect was also confirmed upon arrival in Dakar (A4). This might be due to a positive impact of the reduced metabolic rate due to the chilling on cellular mechanisms to repair somatic damage.

The long-distance shipment to Dakar also significantly affected the rate of operational flies, as already demonstrated [15,19]. Physical injuries caused by vibrations or shocks during transport are probably responsible for this loss. We suggest a new study on the effect of vibration on tsetse flies irradiated pupae during the transport. However, like with the emergence rate, the results observed here seem to improve with time during project implementation. Pagabeleguem et al.[19]observed a rate of operational flies at the ISRA of 35.8± 18.4% for shipments between May 2012 to January 2015 against 51 ± 21% in this study. This increase shows that the overall handling process of flies since its first implementation continuously improved and could probably be improved even more.

### Survival under stress

None of the treatments had an impact on fly survival (*P* > 0.05). However, the mean survival was significantly lower for males emerging from the A1 treatment group of the SAS pupae as compared with the CIRDES pupae (*P* < 0.001), which might be related to differences in the quality of the blood diet and female performance in the two insectaries. During this survival experiment, newly emerged male flies were not offered a blood meal, and their survival only depended on the fat reserves acquired during larval development. The amount of fat reserves is closely related to the quality of the blood and the quantity taken up by the female parent, but also to factors which affect the amount of fat consumed during pupal development (such as temperature) and hence influence the lifespan of the newly emerged fly[32].

The survival of sterile males from the A4 batch at the ISRA was significantly better than survival of the males from the A3 batch at the CIRDES (*P* = 0.006). This suggests that the environmental conditions at the ISRA insectary were more suitable for *G*. *p*. *gambiensis* than at the CIRDES. In this case, blood could not be a determining factor because sterile male flies originated from the same insectary.

## Conclusion

This study highlights the effects of several processes necessary to prepare and transport sterile male tsetse pupae in AW-IPM programs that have an SIT component. Chilling, irradiation and transport all negatively impacted the overal quality of sterile male pupae as revealed by lower emergence rates and lower percentages of operational flies. However, no effect was observed on survival of the emerged male flies. Although the studied processes negatively impact the sterile male yield and quality, longitudinal comparison of data from the Niayes eradication project highlights that sterile male quality improved with time. In order to limit the chilling effect in the insectaries in an attempt to improve the quality of sterile males, the project decided to split the delivery of sterile males into two shipments per week. Further optimizing the quality of the sterile male tsetse could be obtained by further reducing the chilling time and mitigate vibrations during transport. It would be of interest to determine the threshold of chilling duration at which the emergence rate and the rate of operationnal flies can be optimised.

## Supporting information

S1 Table Average percentages of emergence and operational flies depending on the treatment (A0 to A4) and the site where the test was performed. Batches correspond to the number of subsamples of 50 pupae collected from each consignment sent to Dakar.
(DOCX)

S2 Table Summary of the binomial linear mixed effects models for emergence rate. The reference level is CIRDES_A1.
(DOCX)

S3 Table Summary of the binomial linear mixed effects models for the operational rate. The reference level is CIRDES_A1.
(DOCX)

S4 Table Summary of the linear models for mortality rate. The reference level is CIRDES_A1.
(DOCX)
