# Supplementary material for: Chilling, irradiation and transport of male Glossina palpalis gambiensis pupae: Effect on the emergence, flight ability and survival
Source: PLoS One. 2019 May 14;14(5):e0216802. doi: 10.1371/journal.pone.0216802 (PMC6516675; doi:10.1371/journal.pone.0216802)
Supplement: S1 Table — Batches correspond to the number of subsamples of 50 pupae collected from each consignment sent to Dakar. (DOCX) [file pone.0216802.s001.docx]

**S1 Table** . Average percentages of emergence and operational flies depending on the treatment (A0 to A4) and the site where the test was performed. Batches correspond to the number of subsamples of 50 pupae collected from each consignment sent to Dakar.

|  |  |  |  |  |
| --- | --- | --- | --- | --- |
| Site test | Treatment | Nb batches | Mean emergence rate (±SD) | Mean operational rate (±SD) |
| CIRDES | A0 | 116 | 92±8 | 82±13 |
| CIRDES | A1 | 110 | 87±13 | 64±20 |
| CIRDES | A2 | 113 | 85±13 | 45±21 |
| CIRDES | A3 | 114 | 82±17 | 52±21 |
| ISRA | A4 | 468 | 78±15 | 51±21 |
| SAS | A1 | 146 | 86±8 | 82±9 |
| TOTAL |  | 1067 | 83±14 | 60±23 |

NB= number; SD= Standard Deviation, Nb batches=number of biological replicates
