# Supplementary material for: Chilling, irradiation and transport of male Glossina palpalis gambiensis pupae: Effect on the emergence, flight ability and survival
Source: PLoS One. 2019 May 14;14(5):e0216802. doi: 10.1371/journal.pone.0216802 (PMC6516675; doi:10.1371/journal.pone.0216802)
Supplement: S2 Table — The reference level is CIRDES_A1. (DOCX) [file pone.0216802.s002.docx]

**S2 Table**. Summary of the binomial linear mixed effects models for emergence rate. The reference level is CIRDES_A1

| Fixed effects | Estimate | Std. Error | Z value | *P* value |
| --- | --- | --- | --- | --- |
| Intercept | 2.249 | 0.063 | 35.76 | <0.001 |
| CIRDES A0 | 0.585 | 0.065 | 9.06 | <0.001 |
| CIRDES A2 | -0.104 | 0.056 | -1.86 | 0.0635 |
| CIRDES A3 | -0.393 | 0.054 | -7.24 | <0.001 |
| ISRA A4 | -0.887 | 0.045 | -19.51 | <0.001 |
| SAS A1 | -0.43 | 0.059 | -7.24 | <0.001 |
