# Supplementary material for: Chilling, irradiation and transport of male Glossina palpalis gambiensis pupae: Effect on the emergence, flight ability and survival
Source: PLoS One. 2019 May 14;14(5):e0216802. doi: 10.1371/journal.pone.0216802 (PMC6516675; doi:10.1371/journal.pone.0216802)
Supplement: S3 Table — The reference level is CIRDES_A1. (DOCX) [file pone.0216802.s003.docx]

**S3 Table**. Summary of the binomial linear mixed effects models for the operational rate. The reference level is CIRDES_A1.

| Fixed effects | Estimate | Std. Error | Z value | *P* value |
| --- | --- | --- | --- | --- |
| Intercept | 0.784 | 0.053 | 14.74 | <0.001 |
| CIRDES A0 | 1.001 | 0.046 | 21.77 | <0.001 |
| CIRDES A2 | -0.813 | 0.04 | -20.24 | <0.001 |
| CIRDES A3 | -0.542 | 0.04 | -13.55 | <0.001 |
| ISRA A4 | -0.737 | 0.033 | -21.95 | <0.001 |
| SAS A1 | 0.673 | 0.047 | 14.17 | <0.001 |
