# Supplementary material for: Chilling, irradiation and transport of male Glossina palpalis gambiensis pupae: Effect on the emergence, flight ability and survival
Source: PLoS One. 2019 May 14;14(5):e0216802. doi: 10.1371/journal.pone.0216802 (PMC6516675; doi:10.1371/journal.pone.0216802)
Supplement: S4 Table — The reference level is CIRDES_A1. (DOCX) [file pone.0216802.s004.docx]

**S4 Table**. Summary of the linear models for mortality rate. The reference level is CIRDES_A1

| Fixed effects | Estimate | Std. Error | Z value | P value |
| --- | --- | --- | --- | --- |
| Intercept | 4.35 | 0.104 | 41.687 | <0.001 |
| CIRDES A0 | 0.244 | 0.14 | 1.742 | 0.082 |
| CIRDES A2 | 0.023 | 0.149 | 0.154 | 0.8779 |
| CIRDES A3 | 0.183 | 0.147 | 1.24 | 0.2151 |
| ISRA A4 | 0.491 | 0.111 | 4.414 | <0.001 |
| SAS A1 | -2.01 | 0.153 | -13.132 | <0.001 |
